# How culture influences perspective taking: differences in correction, not integration

**DOI:** 10.3389/fnhum.2013.00822

**Published:** 2013-12-02

**Authors:** Shali Wu, Dale J. Barr, Timothy M. Gann, Boaz Keysar

**Affiliations:** ^1^School of Economics and Management, Tsinghua UniversityBeijing, China; ^2^School of Management, Kyung Hee UniversitySeoul, South Korea; ^3^Institute of Neuroscience and Psychology, University of GlasgowGlasgow, UK; ^4^University of California at MercedMerced, CA, USA; ^5^Department of Psychology, The University of ChicagoChicago, IL, USA

**Keywords:** perspective taking, comprehension, cultural differences, ambiguity, reference

## Abstract

Individuals from East Asian (Chinese) backgrounds have been shown to exhibit greater sensitivity to a speaker’s perspective than Western (U.S.) participants when resolving referentially ambiguous expressions. We show that this cultural difference does not reflect better integration of social information during language processing, but rather is the result of *differential correction*: in the earliest moments of referential processing, Chinese participants showed equivalent egocentric interference to Westerners, but managed to suppress the interference earlier and more effectively. A time-series analysis of visual-world eye-tracking data found that the two cultural groups diverged extremely late in processing, between 600 and 1400 ms after the onset of egocentric interference. We suggest that the early moments of referential processing reflect the operation of a universal stratum of processing that provides rapid ambiguity resolution at the cost of accuracy and flexibility. Late components, in contrast, reflect the mapping of outputs from referential processes to decision-making and action planning systems, allowing for a flexibility in responding that is molded by culturally specific demands.

## INTRODUCTION

The human language comprehension system is shaped by informational demands related to communication that are relatively universal, as well as by demands of a more social nature that can vary widely across cultures. On the universal side, spoken language is inherently ambiguous at multiple levels, from lexical processing all the way up to the identification of speech acts and resolution of referential ambiguity. In addition, the speech signal itself is evanescent, requiring language comprehenders to rapidly commit to specific parsing decisions and interpretations. On the culturally specific side, cultures vary in the underlying norms and values that regulate social behavior, including norms for participation in conversational interaction. Do the cultural norms governing language and social interaction impact language processing as immediately and as powerfully as the universal demands for rapid ambiguity resolution? Or do they mainly determine how outputs from relatively universal processes are mapped onto later decisions and actions?

One way of addressing these general questions is to compare language users from different cultures in terms of how they integrate social and linguistic information during the online processing of referring expressions. In this study, we investigated cultural differences in how Chinese vs. Western (U.S.) language users take into account a speaker’s diverging perspective when they resolve ambiguous references such as *the candle*. Referring expressions are of theoretical interest not only because they are ubiquitous in conversation, but also because they require listeners to go beyond the input – an expression such as the *candle* denotes a particular class of object, not any particular individual object, and so listeners must access further information to determine which candle is being spoken about. When speakers and listeners have different visual perspectives, reference resolution will only be consistently successful if listeners take these differences into account. It is also methodologically convenient to study visual perspective taking during reference resolution, because a listener’s eye gaze during the search for a referent provides an external index of the moment-by-moment process of language interpretation (Cooper, 1974; Tanenhaus et al., 1995). The waxing and waning of referential alternatives during processing will be reflected in moment-by-moment changes in the probability distribution of eye gaze over these alternatives.

Studies of perspective taking during reference resolution have experimentally created differences in perspective between speakers and listeners, and monitored listeners’ interpretations as they interpret speakers’ instructions to manipulate objects (Keysar et al., 2000; Nadig and Sedivy, 2002; Hanna et al., 2003). These studies suggest that listeners momentarily experience egocentric interference, with listeners considering “privileged” information that they know is unavailable to the speaker. For example, when searching for a referent for the expression *the candle*, listeners will temporarily consider a candle that is hidden from the speaker’s view, in spite of their knowledge that the speaker does not know about it and therefore could only be referring to another, mutually visible candle (Keysar et al., 2000, 2003). Ultimately, listeners tend to eventually choose the mutually visible candle, although sometimes they may exhibit signs of confusion. For example, listeners frequently ask the speaker to clarify the reference, even though if they took the speaker’s point of view, they would realize the reference was perfectly clear.

Although the basic phenomenon of egocentric interference has been replicated in numerous studies, recent evidence suggests that it might be specific to the Western (European and North American) populations that have been the traditional object of study (Wu and Keysar, 2007). Cultures differ in the extent to which they emphasize the thoughts and beliefs of the individual versus those of the larger group, with cultures of East Asia exhibiting a more “collectivist” character relative to Western cultures, which tend to be more “individualist” in nature (Triandis et al., 1988; Markus and Kitayama, 1991; Ross et al., 2002). Lifelong membership in a particular culture may shape one’s tendency or ability to take another’s perspective into account while comprehending language. If so, then people from East Asian backgrounds should show more reliable and effective perspective taking than Westerners in resolving references.

To test this prediction, Wu and Keysar (2007) conducted an eye-tracking study using the basic visual perspective-taking task of Keysar et al. (2000), comparing the performance of Mandarin-speaking Chinese to English-speaking North Americans (from the U.S.). Each group performed the task in its participants’ native language. Participants played the role of “listener,” sitting across a table from a confederate “director,” with a set of shelves placed between them. The contents of some of the shelves were visible from both sides, while others were hidden from the speaker’s view. The director had a picture of how the objects in the shelves should be arranged, and told the listener which objects to move and where to move them. Embedded within the interaction were certain pre-scripted test instructions designed to be ambiguous from the listener’s perspective, in that they could refer either to a mutually visible “target” object, or a privileged “competitor” object that was visible only to the listener. For example, in one such instruction the director told the listener to “move the candle to the top row,” in a context where the listener saw two identical candles, only one of which was visible to the speaker. Listeners’ eyes were tracked as they interpreted these test instructions. To provide a baseline, in a control condition, the competitor object was replaced with a non-competitor (an object that did not match the description of the target, such as a toy truck for the “candle” instruction). Egocentric interference would lead to an elevated probability of looking at the hidden competitor (candle) relative to the hidden non-competitor (toy truck), as well as in a delayed latency to fixate on the competitor.

Wu and Keysar (2007) found that while Western participants showed the typical pattern of strong egocentric interference, Chinese participants showed virtually no interference. Unlike their American counterparts, Chinese participants were far less likely to fixate on privileged objects or to ask the speaker to clarify a reference that was ambiguous from their own perspective. In short, the Chinese participants were much more effective overall at taking the speaker’s perspective into account.

How might these cultural differences be explained in terms of underlying cognitive processing? Wu and Keysar (2007) measured egocentric interference in terms of first fixation latency and fixation duration, measures that can detect overall differences between groups, but that do not provide information about when such differences might emerge. To gain further insight into the underlying processes, we reanalyzed the data from Wu and Keysar (2007) using a more time-sensitive analysis in order to investigate the time-course of these cultural differences. Our analysis sought to test whether cultural differences emerged early or late relative to the onset of referential processing. On the one hand, cultural differences in egocentric interference may be present from the earliest moments of referential processing, suggesting that Chinese are able to more effectively use information about perspective to constrain the online processing of referring expression. On the other hand, it is possible that cultural differences emerge late, with both groups showing similar levels of egocentric interference early on, and only diverging later. This latter pattern would imply that the earliest moments of processing are unaffected by social information, and are driven largely by egocentric heuristics that enable rapid ambiguity resolution. Under this view, cultural differences would emerge late because participants from a Chinese background would be faster and more effective than Westerners at suppressing the pragmatically inappropriate information. In other words, cultural differences would not reflect differences in the ability to integrate social information into language processing, but instead would reflect differences in how listeners connect the outcome of basic referential processes to further thought and action.

**FIGURE 1:**
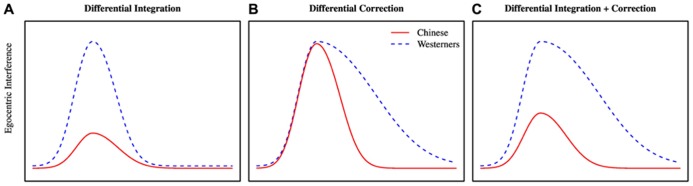
**Predictions of differential integration (A), differential correction **(B)**, and differential integration plus correction (C)**.

Having laid out these possibilities in general terms, let us now consider in more detail the nature of the analysis, the possible outcomes, and their implications for theories of language processing and social cognition. Our analysis focused on the temporal profile of egocentric interference across the two cultural groups. We define egocentric interference as the difference in the likelihood of gazing at a hidden competitor (e.g., candle) versus gazing at a hidden non-competitor (e.g., toy truck). Note that we expect interference to show a curvilinear effect over time as shown by the curves in **Figure 1**, climbing from zero up to a peak from which it will eventually drop (as the listener will ultimately ignore the competitor and select the target).

Based on previous literature, we identify three different effect profiles that would be consistent with three different theoretical accounts. The first account, which we term the *differential integration* account, assumes that the cultural difference reflects the enhanced ability of Chinese to integrate information about the speaker’s perspective with incoming linguistic information. This account would be consistent with constraint-based models of perspective use in language comprehension (Nadig and Sedivy, 2002; Hanna et al., 2003), as these models assume that information about a speaker’s perspective is one of many cues that are simultaneously and interactively integrated during processing. Critically, the account does not differentiate between different types of cues, assuming that any available cue can influence any level of processing from its earliest moments, regardless of its source (e.g., whether it is derived from the unfolding syntax or semantics of the utterance or from situational pragmatics); the influence of a given cue depends only on its salience and reliability. Under this view, the shared perspective between the speaker and listener is a more salient and reliable cue for Chinese than for Westerners. Thus, Chinese should show less egocentric interference than Westerners from the earliest moments of referential processing – in other words, the onset of the cultural difference should be simultaneous with the onset of the overall effect of egocentric interference (**Figure 1A**).

An alternative possibility is suggested by the autonomous activation hypothesis of Barr (2008) which, in contrast to constraint-based accounts, assumes that information about a speaker’s perspective is a kind of situational cue that influences comprehension through anticipatory or post-lexical decision processing, but is not integrated into online lexical processing. Anticipatory processing refers to those steps taken by the listener in preparation for a referring expression, such as increasing attention to shared (mutually visible) objects. Barr (2008) found that comprehenders strongly *anticipated* that speakers would refer to referential candidates that were shared with the speaker, as evidenced by a higher probability of fixating shared than privileged objects. However, supporting autonomous activation, while interpreting the referring expression, listeners did not show any less interference from privileged than from shared competitors: the probability of gazing at a privileged competitor increased from its (lower) baseline at the same rate as the increase in probability for a shared competitor. Strikingly, in one experiment Barr (2008) found that unlike information about the speaker’s perspective, listeners could very efficiently integrate contextual constraints derived from verb semantics. Based on these findings, Barr (2008) argued that lexical processing is encapsulated from high-level information about a speaker’s perspective, and perhaps from other kinds of situational information, but is not strictly modular in the sense of being completely cognitively impenetrable.

The autonomous activation account would predict that Chinese participants might be more sensitive overall to a speaker’s perspective, but without showing any greater ability to integrate this information with the linguistic input. Under this view, they should experience comparable levels of egocentric interference to Westerners, at least during the earliest moments of comprehension. In the current paradigm, this difference would be expressed as *differential correction*: Chinese participants would not initially experience less egocentric interference, but would be faster and more effective at suppressing this interference than Westerners (**Figure 1B**)^1^. Of course, the integration and correction accounts are not mutually exclusive. A third possibility would be that the groups differ in both integration and correction, such that not only do Chinese participants experience lower interference from the earliest moments of comprehension, but they also are more efficient at suppressing this interference (**Figure 1C**). This pattern would be consistent with constraint-based models.

## MATERIALS AND METHODS

Additional details regarding experimental and data collection procedures are available in the original report (Wu and Keysar, 2007).

Our analyses considered looks to the competitor/non-competitor object from 250 ms after the onset of the critical word (e.g., the word “candle” in the phrase “move the candle…”) until 3000 ms. Observations for a given trial were terminated when listeners touched the target. These points varied from trial to trial, with a median of 3306 ms (2808 vs. 3844 for Chinese vs. U.S. participants, respectively), and a standard deviation of 4729 ms. For those trials that were terminated before 3000 ms, we replaced the missing frames with 0 s (representing the absence of a look to the competitor/non-competitor object).

Our goal was to test whether there was a time-lag between the onset of egocentric interference and the onset of cultural differences. To give an overview of our analysis method, we applied the *cluster randomization* method that has become popular in neuroimaging for determining the spatial and temporal extent of experimentally induced effects (Bullmore et al., 1999; Maris and Oostenveld, 2007; for prior adaptation of the solution to the analysis of visual-world data, see Barr et al., 2013). This approach is attractive for localizing effects in time in a visual-world study because it takes advantage of temporal correlations among adjacent data points to overcome the problem of multiple comparisons. The approach proceeds as follows. First, a significance test is performed at each time slice for a given effect (e.g., main effect or interaction). Then, “clusters” are defined by identifying adjacent time slices where the effect reaches significance, and where all effects are in the same direction. For example, consider tests performed at six subsequent time slices, *t*_1_, *t*_2_, *t*_3_, *t*_4_, *t*_5_, and *t*_6_, with tests significant at the 0.05 level only at *t*_2_, *t*_3_, *t*_5_, and *t*_6_. If *t*_2_ and *t*_3_ have effects in the same direction, then they form a cluster; likewise, if *t*_5_ and *t*_6_ are in the same direction, they also form a cluster. There are two separate clusters rather than a single one because of the intervening non-significant test at *t*_4_. Once the clusters have been identified, a “cluster mass statistic” is calculated for each one, typically the sum of all of the individual test statistics (e.g., *t* values) for that cluster. One obtains a null-hypothesis distribution for this cluster mass statistic through randomization (permutation tests); i.e., by randomly shuffling the condition labels across trials to create a large number of new datasets, repeating the above procedure on these datasets, and then storing the maximum obtained cluster mass statistic for each one. Finding a significant cluster between *t*_i_ and *t*_j_ with, say, 1000 additional randomized datasets and *p* = 0.002 means that the cluster mass statistic for the original data was matched or exceeded in only 2 of the 1000 randomly created datasets.

We did this procedure twice, once to test for the main effect of Competition (competitor vs. non-competitor, e.g., egocentric interference), and once to test for the Culture-by-Competition interaction. The cluster randomization procedure provides only *p*-values; however, we were also interested in defining confidence limits for our effects. To obtain these confidence limits we used bootstrapping (details below). The remainder of this section provide further technical details regarding how these analyses were implemented.

**FIGURE 2:**
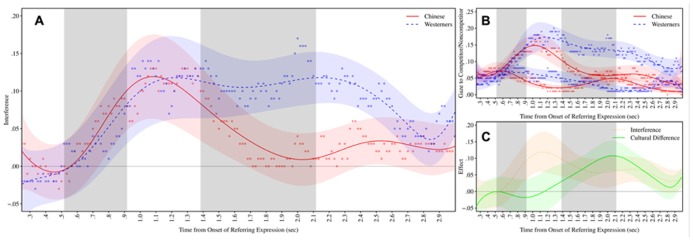
**Time plots of eye-tracking data, showing (A) egocentric interference (competitor minus non-competitor); **(B)** raw probabilities of gazing at the competitor (higher red/blue lines) and non-competitor (lower red/blue lines); and **(C)** time-course of egocentric interference (main effect) against the cultural difference in interference (Culture-by-Competition interaction).** Observed data (points) and model predictions (curves), with the colored bands surrounding each curve representing ±1 SE, and the shaded bars representing 95% confidence intervals for effect onsets. The leftmost bar in each plot corresponds to onset of the competition effect, while the rightmost bar reflects the onset of cultural effects.

Rather than comparing the observed probabilities at each time point, we fit a time-series model to the data and compared predictions from the model, following Barr et al. (2013). The time-series model smooths the data over time, thus minimizing noise and facilitating the detection of clusters (see **Figure 2B**). In the model, time was represented as a 7th order polynomial. We determined the order of the polynomial using a model search procedure, in which we calculated the Akaike Information Criterion (AIC) value for all models ranging from a 3rd order to a 16th order polynomial, and then selected the model with the lowest AIC, which was a 7th order polynomial. This was done on the grand-averaged data (i.e., without any predictors for Competition or Culture) so as not to bias the cluster randomization procedure.

Logistic regression models were fit to the data using the multinom() procedure of the nnet package (Venables and Ripley, 2002) of R statistical software (R Core Team, 2013), treating the outcome for each sample as binary. The cluster randomization procedure was performed twice, once treating subjects as random and items as fixed (*p*_1_), and once treating items as random and subjects as fixed (*p*_2_). For simplicity, we describe the procedure treating subjects as random factors. In addition to the parameter estimates from a fit to the original data, we created 999 additional data sets by randomly permuting the condition labels (competitor vs. control) independently for each unit (subject or item). To obtain orthogonality between the main effects and the interaction, the relabeling followed a “synchronized” permutation logic (Pesarin, 2001). For a given culture group, a permutation was created by randomly choosing, with equal probability, whether or not to block-exchange all competitor and non-competitor labels for each subject. The same number of exchanges was then performed for the other culture group (with the units undergoing the exchange chosen at random). The “synchronization” of exchanges across groups (i.e., ensuring that the same number of exchanges of Competition occurs at each of the two levels of Group) ensures that the tests for the main effects and interaction are orthogonal (Pesarin, 2001). The parameter estimates for the model fit to each of these data sets were stored as a row in the matrix.

After all datasets were created, we then calculated the predicted log odds of a gaze to the competitor/non-competitor object at each 1/60 of a second (i.e., for each frame of data sampled at 60 Hz), deriving main effects and interactions at each time point. The predicted effects for each of the 1000 datasets (including the original) were stored as separate rows in a matrix. The *p*-value for each effect (main effect of competition or interaction) at each time point was given as the number of rows in the effect matrix exceeding the original value divided by the number of rows in the matrix. Then, we identified clusters by grouping together all temporally adjacent time-frames where the effect reached significance. A cluster mass statistic (Bullmore et al., 1999) was calculated for each cluster by summing together the negative (natural) logarithm of each *p*-value belonging to the cluster, such that smaller *p*-values would contribute to a larger cluster mass statistic; for example, for 0.05 the negative log is 3, and for 0.0001 it is 9.21. This cluster mass statistic was calculated for each cluster in the original data. Then, a null-hypothesis distribution for the statistic was derived by treating each permuted data set as if it was the “original” data, calculating *p*-values and cluster mass statistics in the manner described above, and storing the maximum observed statistic for each permutation. This allowed us to identify the onset of the first significant cluster for both the main effect as well as for the interaction.

To obtain confidence limits, we repeated the complete analysis described above for 999 bootstrapped versions of the data set, wherein we sampled subjects with replacement from each group at random. If for a given bootstrapped dataset, no significant cluster for the main effect or interaction was detected at α = 0.05, the α level was progressively lowered until a cluster was detected, stopping at α = 0.2. Although the confidence limits derived from bootstrapping provide useful information, the main inferential focus is on the results of the cluster randomization on the original data.

## RESULTS

The time-course data appear in **Figure 2**. Note that 0 ms does not correspond to the onset of the utterance (e.g., “move” in “move the candle”), but to the onset of the referring expression within the utterance (e.g., “candle”). Thus any differences in timing between groups cannot be attributed to possible linguistic differences between Chinese and English in the duration of the material preceding the referring expression.

The cluster randomization procedure detected significant overall egocentric interference from 750 to 2800 ms, with the 95% confidence interval for the onset of interference ranging from 517 to 917 ms (*p*_1_ < 0.001, *p*_2_ = 0.003)^2^. As **Figure 2** clearly shows, there was a large time-lag between the onset of egocentric interference and the onset of a cultural difference in this interference (as given by a Culture-by-Competition interaction). There was little evidence that Chinese participants experienced any less interference than U.S. participants until 1767 ms, approximately 1000 ms after the onset of interference. The Culture-by-Competition interaction was significant from 1767 to 2483 ms (*p*_1_ = 0.009, *p*_2_ = 0.049), with the 95% confidence interval for the onset ranging from 1383 to 2117 ms. Note that there was no overlap between the confidence interval for the onset of the cultural difference (1383–2117) with that for the onset of egocentric interference (517–917). Furthermore, we directly computed the delay between the onsets for each bootstrapped sample, which yielded a 95% confidence interval for the lag between 600 and 1400 ms.

## DISCUSSION

Overall, our findings support the hypothesis that language users from different cultures share a common stratum of referential processing, with cultural variation in how the products of these early referential processes are used in the higher-level processes governing thought and action. Specifically, whereas neither Chinese nor Western participants were able to integrate the situational cue of the speaker’s perspective into lexical processing, Chinese participants were better able to suppress the interference.

Could our findings of common interference and differential correction be alternatively explained in terms of linguistic differences between Mandarin Chinese and English? One potentially relevant difference is that Mandarin lacks definite marking, such that the Mandarin version of the English expression “move the candle” might be glossed in English as “move candle.” It might be argued that the Chinese participants were interpreting the descriptions as if the speaker had said, “move any candle.” This would indeed predict that the Chinese participants would experience less interference than the U.S. participants because they would not need to decide between the two possible referents, but could pick either one. However, if this were the case, then Chinese participants should have shown a stronger tendency than U.S. participants to move the hidden candle, since any candle would suffice. However, the data showed the exact opposite. While the U.S. participants sometimes moved the occluded candle, the Chinese participants never did.

One possible concern might be that the later correction for Chinese participants reflects shorter referring expressions in Chinese, or more rapid speech when the confederate spoke Chinese. Although we lack the data to directly address this question, the overall patterns shown in **Figure 2** make this explanation seem unlikely. First, if the earlier correction occurred because the Chinese expressions were briefer or spoken more rapidly, then not only would the correction process take place earlier, but so would the egocentric interference; specifically, the initial rising slope of the curve should have been much steeper for the Chinese group than for the Western group, and should have reached its peak much earlier. However, egocentric interference seems to rise at similar rates for both groups, and both seem to initially reach their maximum values at roughly the same time (1000–1200 ms). Second, whereas the correction process seems to begin at around 1000 ms for the Chinese group, it seems delayed until about 2200 ms for the American group. This is far too great of a disparity to be explained by differences in the spoken expressions, given that expressions in these types of experiments typically last no more than 1 s. Finally, the groups differ not only in the timing of the correction, but also in the *efficacy* of the correction, with a sudden sharp decline for the Chinese group, and more of a lingering pattern for the Western group. Thus, these patterns seem less likely to be driven by differences in the stimuli, and more likely to reflect true cultural differences in linguistic interpretation.

Constraint-based views would have difficulty accounting for the extreme delay in the emergence of cultural differences relative to the onset of egocentric interference. If, as constraint-based views predict, language users can integrate perspective information from the earliest moments of processing, and Chinese participants attend more strongly to the shared perspective than Westerners, then Chinese participants should have shown less egocentric interference from the very earliest moments of processing. Our view, then, is that despite attending more strongly to shared information, Chinese participants are no better at integrating it into referential processing. However, an alternative view must be considered, which is that perhaps the late emergence does not reflect a standalone correction process, but simply reflects delayed activation of shared information relative to other kinds of information. Under this view, had the shared knowledge become activated earlier, perhaps we would have seen its effects earlier in processing. However, it is unclear what would account for the delayed activation of shared knowledge within the current paradigm. For one, in the current experimental situation, listeners knew well before hearing the referring expression which items their partner could see and which they could not see. In other words, information about what was shared was available to participants even before any referential information became available. It is therefore not clear why listeners would wait for a referring expression to activate the shared knowledge, rather than using it to predict potential referents in advance. It is not possible to tell whether listeners in fact made such predictions, because this requires comparing shared to privileged objects, and our analysis only considered privileged objects. However, experiments using a similar setup have found that in the interval preceding the onset of the referring expression, listeners are more likely to look at shared objects (Keysar et al., 2000). Furthermore, recent experiments including conditions where competitors/non-competitors are shared show that listeners spontaneously access shared knowledge prior to the onset of referring expressions, but are unable to integrate this information into early referential processes (Barr, 2008). Specifically, listeners attend less overall to privileged objects than to shared objects, but nonetheless experience similar levels of interference from competitors regardless of whether they are shared or not. It would be of interest to repeat these experiments with East Asian participants. Our account predicts greater access to shared knowledge among East Asians, but without any reduction in the size of the interference produced by competitors.

Our view that information about perspective is involved in correction is consistent with an anchoring and adjustment view of perspective taking (Keysar et al., 2000), in which listeners anchor interpretation in their own perspectives, and use information about the speaker’s perspective to incrementally adjust away from the anchor. However, distinct from Keysar et al.’s (2000) original formulation, our findings, together with those of Barr (2008), suggest that listeners do not strategically “anchor” in their own egocentric perspective as a kind of reasoning heuristic; rather, their anchoring is forced upon them by the autonomous activation of referents by low-level interpretation processes that are blind to information about the speaker’s perspective (Barr, 2008). Under this view, the noted egocentrism of listeners might be best characterized as a form of “mental contamination” – i.e., the result of rapid, automatic processes that are beyond control and possibly even awareness (Wilson and Brekke, 1994).

Consistent with the use of common ground in correction, other research shows that perspective taking involves cognitive effort (Rossnagel, 2000; Brown-Schmidt, 2009; Nilsen and Graham, 2009; Lin et al., 2010), and recent neuroimaging evidence suggests a role for the medial pre-frontal cortex in the adjustment process (Tamir and Mitchell, 2010). Furthermore, the correction account is also consistent with dual process views of perspective taking, which assume that social judgments reflect the combination of both efficient but inflexible processing that uses limited information and more flexible but effortful processing that can draw upon a broader set of information (Apperly and Butterfill, 2009). However, the current data offer no insight into why the adjustment process might differ across the groups. One possibility, consistent with the collectivist vs. individualist distinction, is that information about a speaker’s perspective is simply more available to people from a collectivist background, since their cultures require greater attunement to one anothers’ knowledge. Another is that perhaps Chinese participants are more motivated to perform the task “correctly” due to heightened concerns about self-presentation. A further possibility is that membership in a Chinese culture, where self-control is valued, results in better executive control abilities. This explanation is supported by research that finds enhanced executive control abilities among Chinese as opposed to North American children (Sabbagh et al., 2006), who nonetheless showed comparable performance on a belief reasoning task. As we have argued here and elsewhere (Keysar et al., 2003; Barr, 2008) listeners’ difficulty in identifying the intended referent in conversational perspective-taking tasks is unlikely to be the result of a failure to have the appropriate beliefs about what is shared with the speaker. Instead, it seems to reflect difficulty using this information to constrain the processing of the linguistic input. To the extent that early referential processes are not guided by beliefs about the speaker, these processes will boost activation of referents that are pragmatically implausible, even in spite of correct and accessible representations of shared knowledge. Because suppressing this knowledge will involve executive control, it is here where we would expect to see strong individual (and cultural) differences. Although in this respect our view is consistent with Sabbagh et al.’s (2006) developmental findings, it is important to note that it is not yet known whether the differences in executive function that Sabbagh et al. (2006) noted extend into adulthood.

Whatever the explanation for the cultural differences, a recent study suggests that it might be possible to induce cultural effects through priming. Luk et al. (2012) replicated Wu and Keysar’s (2007) study but with Chinese-Westerner bi-cultural individuals. Participants primed by images from Western culture committed more egocentric errors on the perspective-taking task relative to participants who were primed by images from Chinese culture. The fact that cultural differences can be situationally induced in bicultural individuals suggests that they arise from flexible modes of processing. This flexibility is consistent with our explanation of such differences in terms of differential correction – it would seem easier to override a deliberative and effortful correction process than an integration process that is largely routinized and automatic.

In sum, our data suggest that people from different cultures share a common core of ambiguity resolution processes, but differ in how the output from these processes is linked to higher-level systems governing thought and action. The two cultures we have studied show systematic differences in how they prioritize the individual vs. the social (Triandis et al., 1988; Markus and Kitayama, 1991; Ross et al., 2002). Finding equivalent interference from privileged information in spite of such differences suggests that such egocentrism might be a universal consequence of rapid ambiguity resolution during spoken language comprehension.

## Conflict of Interest Statement

The authors declare that the research was conducted in the absence of any commercial or financial relationships that could be construed as a potential conflict of interest.

## Footnotes

1 Unlike Barr (2008), the current study does not offer the possibility of determining whether listeners were attentionally biased toward shared referential alternatives before hearing the referring expression. This would require additional conditions in which the competitor or non-competitor would be shared with the speaker.

2 Although an onset of 750 ms is quite late relative to typical visual-world studies (250–350 ms), this is not surprising given that our paradigm presented participants with a more demanding search task than in a typical study. Whereas a typical grid in Wu and Keysar (2007) contained nine alternatives appearing in any of 16 possible locations, a typical visual-world task presents no more than four referential alternatives in fixed locations (Huettig et al., 2011).
